# 5-Arylidene-1,3-dialkylbarbituric acid derivatives as efficient corrosion inhibitors for carbon steel in molar hydrochloric acid solution

**DOI:** 10.1039/d2ra00696k

**Published:** 2022-04-06

**Authors:** Khaled M. Abd El-Khalek, Kamal Shalabi, Mohamed A. Ismail, Abd El-Aziz S. Fouda

**Affiliations:** Department of Chemistry, Faculty of Science, Mansoura University Mansoura-35516 Egypt asfouda@mans.edu.eg

## Abstract

The inhibiting impact of two ecofriendly 5-arylidene barbituric acid derivatives (5-ABA), namely 5-(3,4-dimethoxybenzylidene)-1,3-dimethylpyrimidine-2,4,6(1*H*,3*H*,5*H*)-trione (inhibitor I, 3a) and 5-(3,4-dimethoxybenzylidene)-1,3-diethyl-2-thioxodihydropyrimidine-4,6(1*H*,5*H*)-dione (inhibitor II, 3b), in 1 M HCl on the corrosion of carbon steel has been examined *via* the weight loss (WL) method, potentiodynamic polarization (PP), electrochemical impedance spectroscopy (EIS), and electrochemical frequency modulation (EFM) tests. In addition, DFT calculations and MC simulations were used to study the relationship between the inhibitor structure and its inhibition performance. The attained outcomes exhibit that the investigated compounds are excellent inhibitors and their inhibition efficiency (%IE) increases with the increase in the concentration and temperature. The adsorption of 5-arylidene barbituric acid on the C-steel surface was found to follow the Langmuir adsorption isotherm. The adsorption process of the investigated compounds is spontaneous and considered as the chemisorption type. The PP curves revealed that 5-arylidene barbituric acid derivatives are mixed-type inhibitors. Moreover, the EIS results confirmed the adsorption of 5-arylidene barbituric acid derivatives on the C-steel surface by increasing the charge transfer resistance (*R*_ct_) values. The %IE of the inhibitors (II & I) reached 92.8% and 86.6% at a concentration of 21 × 10^−6^ M, according to the WL method. The surface analysis of the C-steel surface was confirmed by scanning electron microscopy and energy dispersive X-ray techniques. Finally, the experimental and theoretical results are in good agreement.

## Abstract 2

The inhibiting impact of two 5-arylidene barbituric acid derivatives on the corrosion of carbon steel has been examined *via* the weight loss method, potentiodynamic polarization, electrochemical impedance spectroscopy, and electrochemical frequency modulation tests.

## Graphical abstract

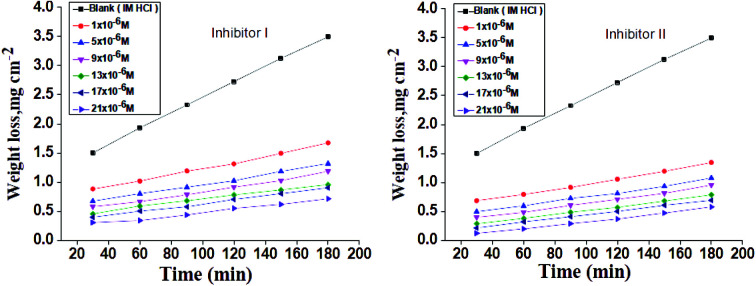

## 1 Introduction

Acidic media are generally applied for the elimination of unwanted scales and corrosion in several industrial procedures. By monitoring metal dissolution due to acidic exposure, inhibitors are commonly applied within these operations.^1^ Nowadays, organic inhibitors show better inhibition of corrosion than inorganic inhibitors.^2^ Organic compounds are a kind of acidic inhibitors including heteroatoms, for example, oxygen, sulfur, and nitrogen. Among these, organic inhibitors have several advantages, for instance, not expensive, low poisonousness, high inhibition efficiency, and easy to organize.^3–6^ In general, heterocyclic organic compounds are applied for corrosion inhibition on copper,^7^ aluminum,^8–10^ iron,^11–16^ and also other metals^17,18^ within a diverse corrosion media. A review of the literature on acid corrosion inhibitors reveals that they work by adsorbing on the metal's surface. This effect may be caused by electrostatic attraction between the charged metal and the charged inhibitor molecules, (ii) dipole-type interaction between the uncharged electron pairs in the inhibitor and the metal, (iii) electron-interaction with the metal, or (iv) a combination of the aforementioned.^19^ Pyrimidine is a six-membered heterocyclic aromatic chemical molecule with two nitrogen atoms at positions 1 and 3. The chemistry of pyrimidine derivatives is crucial in medicine, agrochemicals, and a variety of biological activities. Numerous well-known commercial medications contain pyrimidine derivatives, such as Uramustine, Piritrexim, Isetionate, Tegafur, Floxuridine, Fluorouracil, Cytarabine, and Methotrexate. Furthermore, the pyrimidine skeleton is found in a wide range of natural products, including nucleic acids, vitamins, enzymes, chlorophyll, haemoglobin, and hormones. A list of organic derivatives utilized as corrosion inhibitors for metals is shown below:

**Table:** 

Pyrimidine derivatives	Sample	Medium	%IE	Ref.
5-Phenyl-1,3,5,6,8-pentahydro-pyrimido[4,5-*d*] pyrimidine-2,4,7-trione (PPD-4), 5-(4-methoxyphenyl)-1,3,5,6,8-pentahydropyrimido[4,5-*d*] pyrimidine-2,4,7-trione (PPD-3), 5-phenyl-1,3,5,6,8-pentahydro-7-thioxo-pyrimido[4,5-*d*]pyrimidine-2,4-dione (PPD-2), and 5-(4-methoxyphenyl)-1,3,5,6,8-pentahydro-7-thioxo-pyrimido[4,5-*d*]pyrimidine-2,4-dione (PPD-1)	Mild steel	1 M HCl	88–97.1% at 400 mg L^−1^	20
(a) 6-Methyl-4-morpholin-4-yl-2-oxo-,2,3,4-tetrahydro-pyrimidine-5-carboxylic-acid-ethyl-ester	Carbon steel	0.5 M HCl	80–86 at 0.25 g L^−1^	21
(b) 6-Methyl-4-morpholin-4-yl-2-thioxo-1,2,3,4-tetrahydro-pyrimidine-5-carboxylic acid ethyl ester	Mild steel	0.5 M HCl	80–86 at 0.25 g L^−1^	21
(c) 6-Methyl-4-morpholin-4-yl-2-oxo-1,2,3,4-tetrahydro-pyrimidine-5-carboxylic acid hydrazide				
(d) 6-Methyl-4-morpholin-4-yl-2-thioxo-1,2,3,4-tetrahydro-pyrimidine-5-carboxylic acid hydrazide				
5-Benzoyl-4-(4-carboxphenyl)-6-phenyl-1,2,3,4-tetrahydro-2-iminopyrimidine, 5-benzoyl-4-tolyl-6-phenyl-1,2,3,4-tetrahydro-2-thioxopyrimidine in 1 M HCl	Stainless steel	1 M HCl	90 at 5 × 10^−3^ M	22
5-Benzoyl-4-(substituted phenyl)-6-phenyl-3,4-dihydropyrimidine-2(1*H*)-(thio)ones in 0.5 M H_2_SO_4_	Stainless steel	0.5 M H_2_SO_4_	92 at 2 × 10^−3^ M	23
(a) 5-(4-Methoxyphenyl)-1,3,5,6,8-pentahydro-7-thioxo-pyrimido[4,5-*d*]pyrimidine-2,4-dione	Mild steel	1 M HCl	97.1–88.0 at 400 ppm	24
(b) 5-Phenyl-1,3,5,6,8-pentahydro-7-thioxo-pyrimido[4,5-*d*]pyrimidine-2,4-dione				
(c) 5-(4-Methoxyphenyl)-1,3,5,6,8-pentahydro-pyrimido[4,5-*d*]pyrimidine-2,4,7-trione				
(d) 5-Phenyl-1,3,5,6,8-pentahydro-pyrimido[4,5-*d*]pyrimidine-2,4,7-trione in HCl				
1-(7-Methyl-5-morpholin-4-yl-thiazolo[4,5-*d*]pyrimidin-2-yl)-hydrazine	Carbon steel	0.5 M H_2_SO_4_	90 at 400 ppm	25
(a) 4,6-Diphenyl-3,4-dihydropyrimidine-2(1*H*)-thione	Carbon steel	1 M H_2_SO_4_	99–98 at 10 mM	26
(b) 4-(4-Methylphenyl)-6-phenyl-3,4-dihydropyrimidine-2(1*H*)-thione				
(c) 4-(4-Methoxy-phenyl)-6-phenyl-3,4-dihydropyrimidine-2(1*H*)-thione				
(a) 4-(4′-Methylphenyl)-6-(phenyl)-3,4-dihydropyrimidine-2(1*H*)-thione	Stainless steel 304	2 M H_2_SO_4_	97.8, 96.2 at 5 mM	27
(b) 4-(4′-Methoxylphenyl)-6-(phenyl)-3,4-dihydro-pyrimidine-2(1*H*)-thione in 2.0 M H_2_SO_4_ (ref. 39) for stainless steel 304				
(3a, MA-1230), (3b, MA-1231) and (3c, MA-1232)	Copper	1 M HNO_3_	90.3–92.1 at 21 μM	28
(i) Ethyl(2-amino-5-methyl[1,2,4]-triazolo[1,5-*a*]pyrimidin-7-yl)acetate	Mild steel	1 M HCl	84, 85, respectively at 10^−3^ M	29
(ii) Ethyl(5-methyl[1,2,4]triazolo[1,5-*a*]pyrimidin-7-yl)-acetate				

The efficacy of the organic compounds including hetero atoms as corrosion inhibitors in acidic solutions for C-steels is well recognized.^30–34^ Pyrimidines and their derivatives are important because they are available in nature, particularly in the nucleobases present in nucleic acids, and many of them have been discovered to be beneficial in chemotherapy.^35^ Currently in use as anticancer, antifungal, and antibacterial medicines are pyrimidine-containing chemotherapeutics.^36^ Furthermore, in HCl and H_2_SO_4_ solutions, several pyrimidine derivatives were found to be efficient corrosion inhibitors for steel.^37^

The purpose of this work is to study the impact of 5-arylidene barbituric acid derivatives as ecofriendly inhibitors for C-steel in 1 M hydrochloric acid solution by applying WL, PP, EIS, and EFM tests. These 5-arylidene 1,3-dialkylbarbituric acid derivatives are less toxic, have large molecular sizes, and contain donating atoms such as N, O, S, benzene ring, and groups such as CH_3_ or C_2_H_5_. In addition, computational studies (*i.e.*, DFT calculation and MC simulations) were undertaken to demonstrate the adsorption sites found in the inhibitor's molecules. These 5-arylidene barbituric acid derivatives have not been reported as corrosion inhibitors for steel in the literature until now.

## 2 Materials and methods

### 2.1 Materials

The chemical configuration of C-steel samples in weight percentage is carbon (0.200%); manganese (0.350%); phosphor (0.024%); chromium; sulfur (0.003%); and balance iron.

### 2.2 Inhibitors

**Scheme 1:**
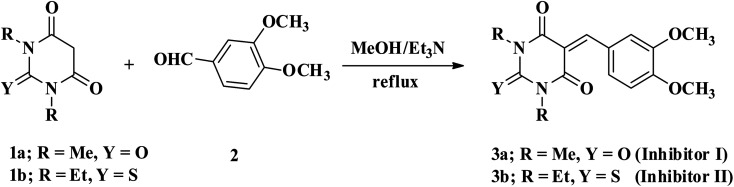
Synthetic routes of 5-aryldine 1,3-dialkylbarbituric acid derivatives.

5-Arylidene barbituric acid derivatives were synthesized as outlined in Scheme 1 (molecular formula, molecular weights, and structures of the studied compounds are presented in Table 1). The detailed information of inhibitor I (3a), including the spectroscopic data has been reported,^38^ mp 229–231 °C, IR (KBr) *ν*′/cm^−1^: 3122, 3004 (sp^2^ C–H stretch), 2947, 2906, 2839 (sp^3^ C–H stretch), 1720, 1651 (CO stretch), 1598, 1556, 1502 (C=C stretch). ^1^H-NMR (CDCl_3_); *δ* 3.39, 3.40 (2 s, 6H; 2× N–CH_3_), 3.97, 3.98 (2s, 6H; 2× OCH_3_), 6.94 (d, *J* = 8.7 Hz, 1H), 7.78 (dd, *J* = 8.7 Hz, 2.1 Hz, 1H), 8.38 (d, *J* = 2.1 Hz, 1H), 8.48 (s, 1H, methine H). Inhibitor II (3b) was prepared by the treatment of 1,3-diethylthiobarbituric acid (1b, 5 mmol) with 3,4-dimethoxybenzaldehyde (2, 5 mmol) in 30 mL methanol at reflux in the presence of triethylamine as the catalyst to afford inhibitor II (3b) in 84% yield, mp 186–187 °C (DMF/EtOH), lit^39^ mp 185–187 °C; IR (KBr) *ν*′/cm^−1^: 3115 (sp^2^ C–H stretch), 2976, 2928, (sp^3^ C–H stretch), 1689 (CO stretch), 1660, 1541, 1502 (C=C stretch), 1381 (C=S stretch) cm^−1^. ^1^H-NMR (DMSO-*d*_6_); *δ* 1.15–1.21 (m, 6H; 2× CH_3_ of ethyl group), 3.81 (s, 3H; OCH_3_), 3.89 (s, 3H; OCH_3_), 4.39–4.44 (m, 4H; 2× CH_2_ of ethyl group), 7.13 (d, *J* = 8.5 Hz, 1H), 7.99 (dd, *J* = 8.5 Hz, 2.0 Hz, 1H), 8.23 (d, *J* = 2.0 Hz, 1H), 8.36 (s, 1H, methine proton). MS (EI) *m*/*z* (rel. int.); 348 (M+, 100).

**Table 1:** The molecular structure of the investigated inhibitors

Inhibitors	Structure/chemical name
Inhibitor (I)	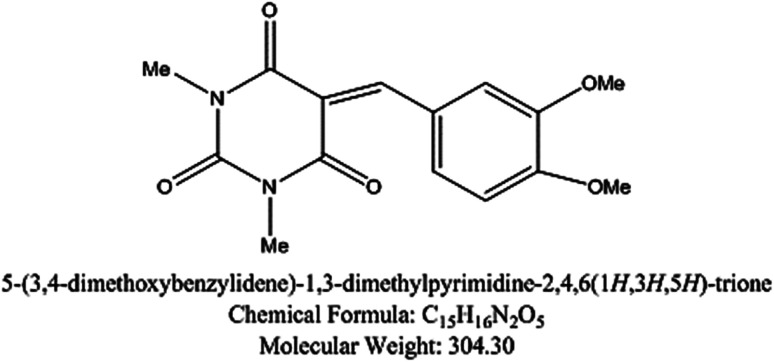
Inhibitor (II)	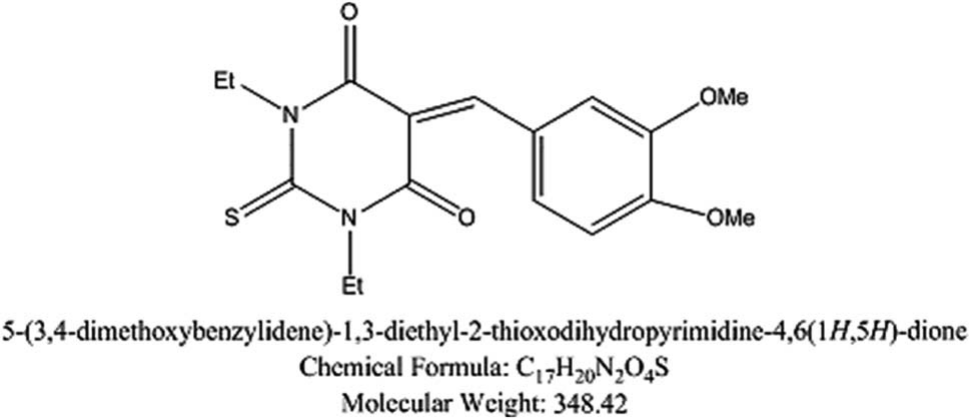

### 2.3 Aqueous solutions

The corrosive solutions, 1 M hydrochloric acid, was prepared by the dilution of analytical grade 37% hydrochloric acid *via* double-distilled water, and the concentration range of the applied inhibitors was 1–21 × 10^−6^ M.

### 2.4 Weight loss (WL) method

Seven identical pieces of C-steel having dimensions 2.5 × 2.0 × 0.06 cm^2^ were polished by abrasive paper (grades 320–1200), then washed by double-distilled water. The pieces were weighted and submerged in a 100 mL beaker, including 100 mL hydrochloric acid without and with diverse concentrations of the examined inhibitors.

Corrosive acid solutions were left open in air. After 30 min intervals, pieces were ejected, cleaned, dried, and then weighed perfectly for 3 h. The *θ* and IE% of the examined inhibitors were calculated from the subsequent equation.^40^1
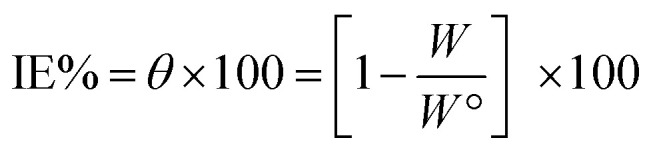
where, *W*° and *W* are the values of the average weight loss without and with the inhibitor, respectively.

### 2.5 Electrochemical techniques

Electrochemical measurements were taken within a traditional three electrode glass cell including saturated calomel electrode (SCE) linked with fine “Luggin capillary, platinum counter electrode, and the working electrode was carbon steel with a square cut shape and surface area of 1.0 × 1.0 cm^2^. PP curves were established by altering the electrode potential automatically from −1000 to 0.0 mV *vs.* OCP with a sweep rate of 0.2 mV s^−1^. The Stern-Geary^41^ definition of corrosion current was achieved *via* deducing on cathodic and anodic Tafel lines to a point that provides log *i*_corr_ and the resulting *E*_corr_ for inhibitor-free acid and to any concentration of the inhibitor. Thereafter, *i*_corr_ can be applied to examine of *θ* and IE% as:2
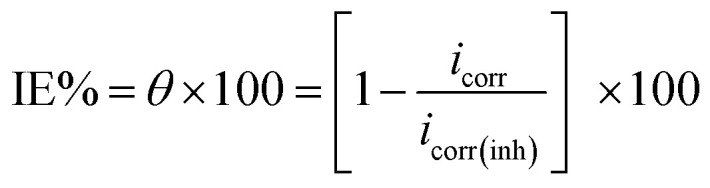
where, *i*_corr(free)_ and *i*_corr(inh)_ are the corrosion current densities in the absence and presence of the inhibitor, respectively.

EIS was applied within the frequency range from 100 kHz to 10 mHz and 5 mV amplitude peak-to-peak at OCP. The *θ* and the IE% achieved from the impedance calculation were assessed through the following equation.3
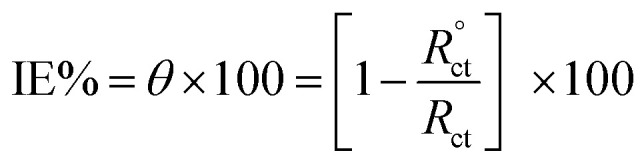
where, 
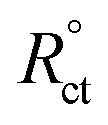
 and *R*_ct_ are the resistance of charge transfer in the absence and existence of the inhibitor, respectively.

EFM tests were accomplished *via* dual frequencies 2 and 5 Hz with a base frequency of 0.1 Hz; consequently, the wave shape repeats subsequently at 1 s. The large peaks located in the intermodulation spectra were utilized to assess the corrosion current density (*i*_corr_), the Tafel slopes (*β*_a_ and *β*_c_), and CF-2 & CF-3;^42,43^ %IE and *θ* were assessed from eqn (2).

All electrochemical experiments were carried out in the solution at 25 ± 1 °C. The potential of the electrode can be permitted until it becomes stable 30 min prior to the start of the measurements. All electrochemical experiments were done at 25 ± 1 °C and accomplished *via* a Gamry (PCI4/750G) Potentiostat/Galvanostat/ZRA. This includes the Gamry Framework for controlling and the Echem Analyst 5.58 software for data analysis and plotting.

### 2.6 DFT calculations and MC simulations

The Dmol3 and adsorption locator modules of Accelrys Inc., USA Materials Studio software V.7.0 were used to perform the DFT calculations and MC simulations. The GGA/BLYP basis set in the aqueous phase was used to optimize the 5-arrylidene barbituric acid derivative molecules.^44^ The following equations were used to compute various quantum parameters such as ionization potential (*I*), electron affinity (*A*), electronegativity (*χ*), global hardness (*η*), and global softness (*σ*).^45^

4 *I* = −*E*_HOMO_

5 *A* = −*E*_LUMO_

6
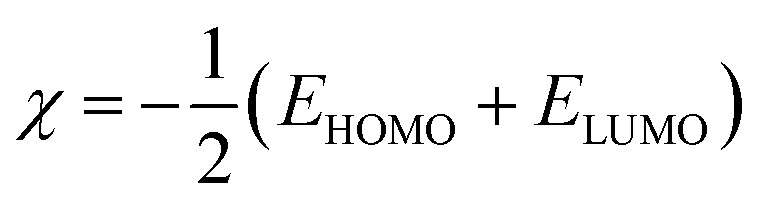
7
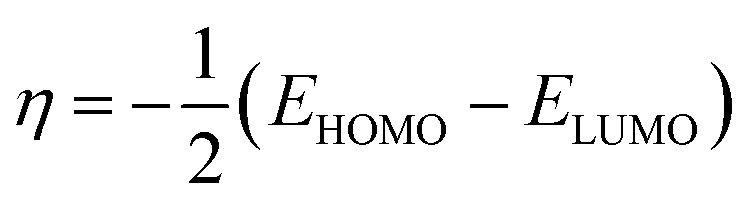
8
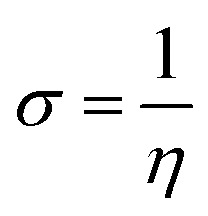


The fraction of electrons transferred (Δ*N*) from the inhibitor molecules to the metallic surface through adsorption can be determined employing eqn (9):9
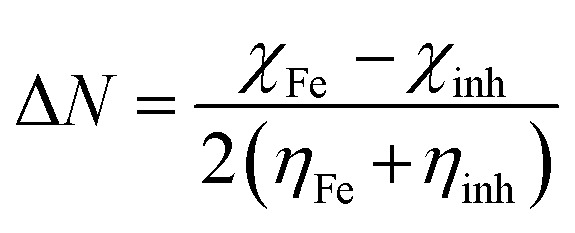
where, “*χ*_Fe_, *χ*_inh_, *η*_Fe_, and *η*_inh_ are the electronegativity and hardness values of Fe and inhibitor molecules, respectively. The value of *χ*_Fe_ is 7.00 eV and *η*_Fe_ is 0.^46^ The adsorption locator discovers the potential adsorption configurations of the 5-arylidene barbituric acid derivative molecules with Monte Carlo searches on the Fe (1 1 0) surface for assessing the inhibition performance of 5-arylidene barbituric acid derivative molecules.^47^ In a simulation box (32.27 Å × 32.27 Å × 50.18 Å) with periodic boundary conditions, the interactions of 5-arylidene barbituric acid molecules and the surface of Fe (110) were accomplished. The energy optimization of 5-arylidene barbituric acid derivatives molecules was implemented by exploiting Forcite classical simulation engine.^48^ The corrosion system in the aqueous media was established *via* the layer builder, and this system involves the optimized 5-arylidene barbituric acid derivatives molecules, Fe (110) surface, and water. For the adsorption capacity simulation of 5-arylidene barbituric acid derivative molecules on the surface of Fe (110), the COMPASS simulation investigation with force field was executed.^49^

### 2.7 Surface examinations

C-Steel samples prior and after immersion in 1 M HCl solutions without and with 21 × 10^−6^ M of compound I and II for 24 h at 25 °C were examined. Then, the C-steel samples were taken and dried. Surface examinations of C-steel samples were achieved by a JEOL JSM-6510 LV for SEM and EDX analysis.

## 3 Results and discussion

### 3.1 Weight loss (WL) method

**Fig. 1:**
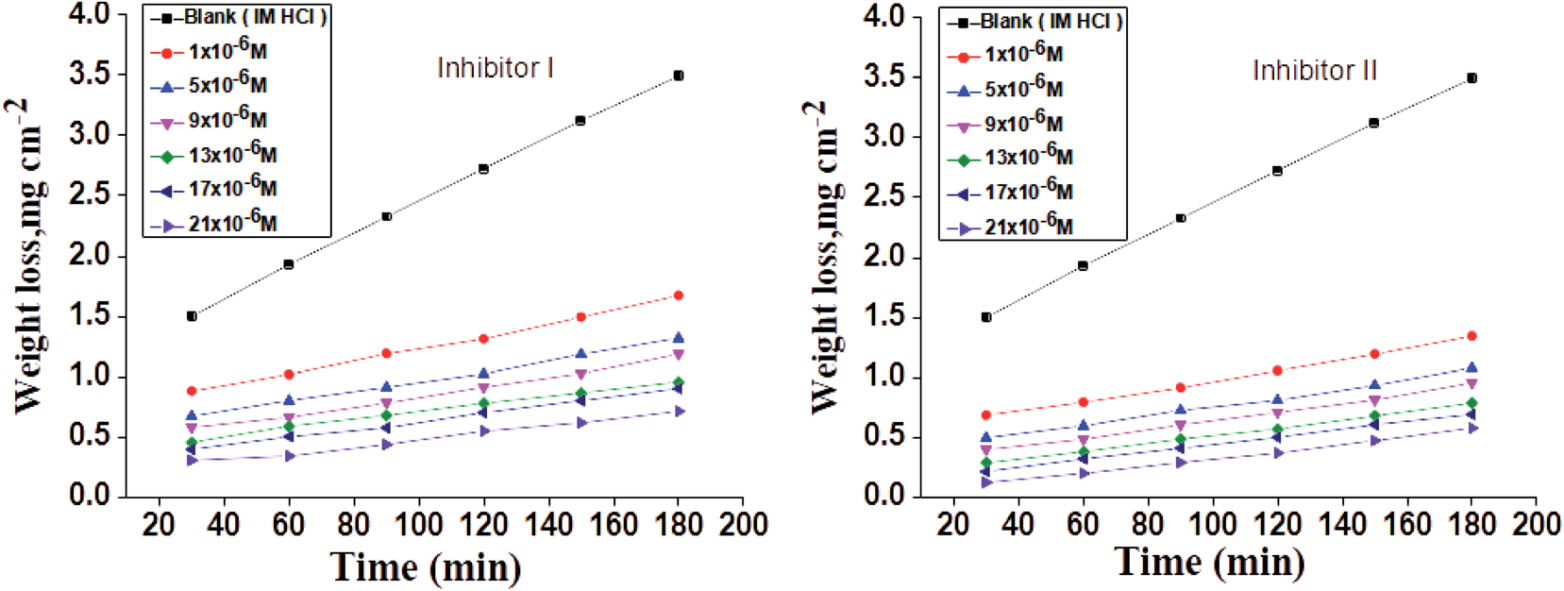
Time-WL curves for C-steel in 1 M HCl in the absence and presence of diverse doses of inhibitors (I and II) at 25 °C.

The WL-time diagrams for the corrosion of C-steel in 1 M hydrochloric solution before and after the addition of diverse concentrations of compounds (I and II) are displayed within Fig. 1. This figure demonstrates that the values of WL for C-steel with 1 M hydrochloric acid solution lies higher than that in inhibitors and the WL decreases as the inhibitor dose increases, which means the strengthening of corroiksion inhibition on increasing the inhibitor concentration, as listed in Table 2. This explains the adsorption of inhibitor molecules on the C-steel surface, *i.e.*, the C-steel surface is shielded from the aqueous media by the creation of a protecting film on this surface.^50,51^ The order of inhibition proficiency for 5-arylidene barbituric acid derivatives achieved from the WL method is inhibitor II > inhibitor I.

**Table 2:** Variation of %IE with altered doses of the investigated compounds at 25 °C from WL measurements at 120 min dipping in 1.0 M HCl

Comp.	Conc. (M)	C.R. (mg cm^−2^ min^−1^)	%IE
Blank	—	0.028 ± 0.0021	—
Inhibitor (I)	1 × 10^−6^	0.015 ± 0.0015	60.8
	5 × 10^−6^	0.012 ± 0.0023	65.5
	9 × 10^−6^	0.010 ± 0.0009	70.9
	13 × 10^−6^	0.008 ± 0.0019	70.9
	17 × 10^−6^	0.007 ± 0.0009	81.0
	21 × 10^−6^	0.006 ± 0.0023	86.6
Inhibitor (II)	1 × 10^−6^	0.012 ± 0.0026	69.1
	5 × 10^−6^	0.009 ± 0.0021	73.9
	9 × 10^−6^	0.007 ± 0.0017	79.2
	13 × 10^−6^	0.006 ± 0.0021	82.5
	17 × 10^−6^	0.005 ± 0.0017	87.6
	21 × 10^−6^	0.003 ± 0.0020	92.8

### 3.2 PP studies

**Fig. 2:**
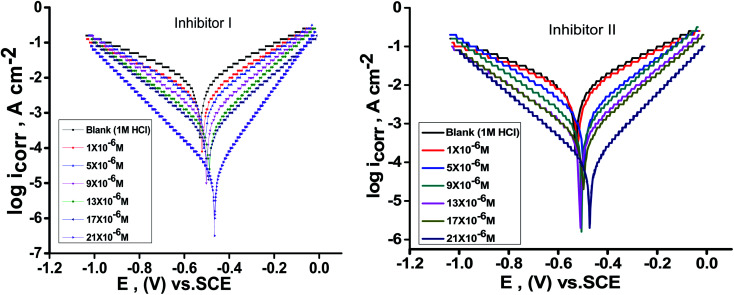
PP diagrams for the dissolution of C-steel in 1 M HCl in the presence and absence of altered doses of inhibitors (I & II) at 25 °C.

Fig. 2 illustrates the Tafel polarization diagrams for C-steel in 1 M hydrochloric acid in the absence and presence of diverse inhibitors doses at 25 °C, respectively. From Fig. 2, it is obvious that anodic metal dissolution and cathodic H_2_ reduction reactions were controlled when these inhibitors were added to 1 M HCl solution. Also, this inhibition was more obvious with increasing doses of inhibitors. Also, these figures show that the cathodic curves give approximately parallel lines, suggesting that the hydrogen discharge reaction lowers, its activation being controlled^52^ by the addition of inhibitors in aggressive medium. The inhibition mode of the anodic process depends on the electrode potential.^52^Table 3 illustrates that *i*_corr_ declines with the addition of the inhibitors and by increasing their doses. Furthermore, *E*_corr_ does not change clearly (70 and 44 mV for I and II, respectively), and this exhibits that these derivatives are considered as mixed-type inhibitors.^53^ Moreover, Tafel slopes [*β*_a_‚ *β*_c_] are almost constant, indicating that the two reactions (*i.e.*, anodic metal dissolution and cathodic hydrogen reduction) were slightly affected without altering the mechanism of dissolution.^54,55^ The order of inhibition efficiency for 5-arylidene barbituric acid derivatives achieved from PP studies is inhibitor II > inhibitor I.

**Table 3:** Corrosion parameters of C-steel electrode in 1 M HCl solution containing altered doses of inhibitors (I & II) at 25 °C from the PP technique

Comp.	Conc., M	−*E*_corr_ mV *vs.* SCE	*i*_corr_ mA cm^−2^	*β*_c_ mV dec^−1^		*β*_a_ mV dec^−1^	C.R. mmy^−1^	*θ*	%IE
1 M HCl	00	531 ± 0.2028	422 ± 0.2028	42 ± 0.2028	22 ± 0.1453		220.6	—	—
Inhibitor (I)	1 × 10^−6^	515 ± 0.2431	246 ± 0.1155	26 ± 0.1421	13 ± 0.2906		130.0	0.459	45.9
	5 × 10^−6^	534 ± 0.2055	234 ± 0.2603	48 ± 0.1535	39 ± 0.2624		96.2	0.634	63.4
	9 × 10^−6^	494 ± 0.1452	214 ± 0.1764	47 ± 0.1214	50 ± 0.2224		75.4	0.791	79.1
	13 × 10^−6^	502 ± 0.1742	174 ± 0.2028	82 ± 0.1121	47 ± 0.2006		62.4	0.814	81.4
	17 × 10^−6^	512 ± 0.2102	101 ± 0.1732	74 ± 0.1074	85 ± 0.2421		45.1	0.834	83.4
	21 × 10^−6^	464 ± 0.2209	58 ± 0.1453	127 ± 0.231	57 ± 0.2028		29.2	0.906	90.6
Inhibitor (II)	1 × 10^−6^	516 ± 0.2119	223 ± 0.1732	20 ± 0.2333	15 ± 0.2082		119.5	0.644	64.4
	5 × 10^−6^	496 ± 0.2010	180 ± 0.2028	46 ± 0.1202	19 ± 0.1732		80.9	0.702	70.2
	9 × 10^−6^	504 ± 0.1753	126 ± 0.2010	45 ± 0.1732	32 ± 0.2082		45.9	0.725	72.5
	13 × 10^−6^	510 ± 0.1613	101 ± 0.1764	75 ± 0.1453	41 ± 0.1764		41.2	0.782	78.2
	17 × 10^−6^	498 ± 0.1421	80 ± 0.1453	67 ± 0.2027	39 ± 0.1154		36.6	0.837	83.7
	21 × 10^−6^	472 ± 0.1253	45 ± 0.1732	112 ± 0.233	67 ± 0.1245		20.9	0.924	92.4

### 3.3. EIS studies

**Fig. 3:**
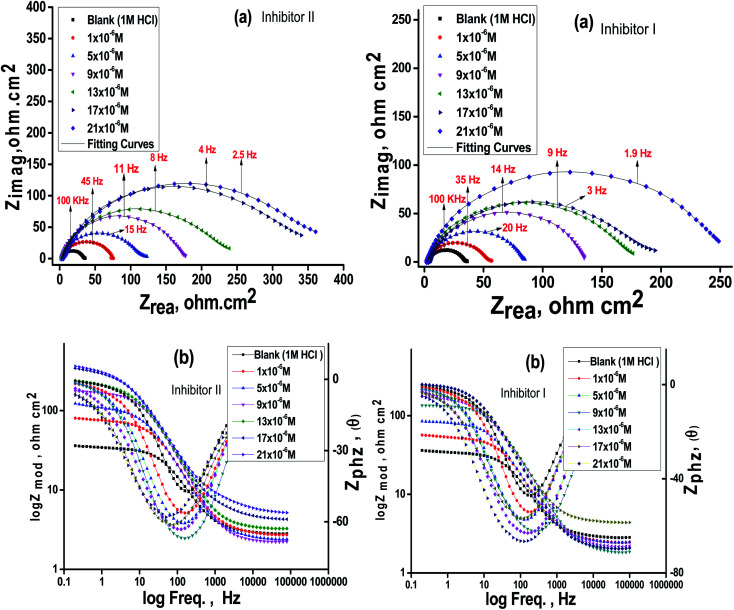
Nyquist (a) and Bode (b) plots for C-steel in 1 M HCl at altered doses of the inhibitors (I & II) at 25 °C.

**Fig. 4:**
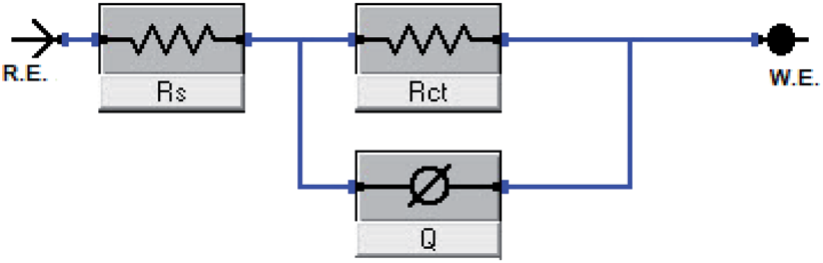
Electrical equivalent circuit model utilized to fit the results of impedance.

The impact of the dose of the inhibitor on the impedance of C-steel in 1 M HCl at 25 °C is shown in Fig. 3a and b. The curves showed identical Nyquist curves for C-steel in the presence of diverse doses of inhibitors (I & II). The presence of a single semi-circle displayed the single charge transfer procedure through dissolution, which is unaltered in the presence of inhibitors. Deviations from the ideal circular form frequently signal the frequency dispersal of impedance interfacial, which occurs because of impurities, surface coarseness, grain limits, dislocations, forming of porous layers, and adsorption of derivatives, which is also homogenized on the surface of the electrode.^56,57^ The observation of these data detected from all the impedance graphs contains a large capacitive circle by only time constant of capacitance with the Bode-phase graphs (Fig. 3b). In the Bode diagram (Fig. 3b), it can be seen that the impedance response of C-steel in HCl solution shows a significant change after inhibitor addition, indicating that the electrode impedance increases with increasing inhibitor doses. From the Bode graph, it may be observed that the phase angle does not exceed 90 °C. The electrical equivalent circuit is displayed in Fig. 4 and it is applied for examining the impedance data. This circuit involves *R*_ct_, *C*_dl_, and also the solution resistance (*R*_s_). Excellent fit through this model can be gained through the experimental data. The EIS outcomes in Table 4 distinguished that the *C*_dl_ values decline and the *R*_ct_ values increase by increasing the doses of the inhibitors. This is due to the exchange of the adsorbed water molecules with the inhibitor molecules on the surface of the metal, decreasing the metal dissolution reaction.^58,59^ The decrease in *C*_dl_ can be caused by a drop in the local dielectric constant and/or a rise in the thickness of the double electrical layer, which suggests that the inhibitor molecules function through adsorption at the metal and solution interface.^60^ It is also worth noting that the “*n*” values increases as the inhibitor doses increase. This can be explained by the reduction in the surface heterogeneity caused by inhibitor molecules adsorbed on the C-steel surface. The precision of fitting outputs was assessed using a chi-square test for goodness of fit; the tiny chi-square values (Table 4) obtained for all the outcomes suggest that the fitted results are very close to the experimental findings. The %IE obtained from the EIS studies are close to those inferred from the PP studies. The order of %IE for 5-arrylidene barbituric acid derivatives achieved from the EIS studies is inhibitor II > inhibitor I.

**Table 4:** EIS data of C-steel in 1 M HCl and in the presence of altered doses of the investigated inhibitors (I & II) at 25 °C

Comp.	Conc., M	*C*_dl_, μF cm^−2^	*R*_ct_, Ω cm^2^	*n*	*θ*	IE%	*χ* ^2^
1 M HCl	00	117.9 ± 0.2333	31.8 ± 0.1764	0.84 ± 0.01	—	—	0.002
Inhibitor (I)	1 × 10^−6^	93.7 ± 0.2145	51.6 ± 0.1453	0.85 ± 0.02	0.386	38.6	0.004
	5 × 10^−5^	88.9 ± 0.1453	81.6 ± 0.2028	0.85 ± 0.02	0.613	61.3	0.003
	9 × 10^−5^	81.2 ± 0.1732	134.1 ± 0.2309	0.87 ± 0.01	0.764	76.4	0.005
	13 × 10^−5^	78.9 ± 0.1245	178.3 ± 0.1732	0.86 ± 0.01	0.822	82.2	0.001
	17 × 10^−5^	63.6 ± 0.1178	197.3 ± 0.2028	0.87 ± 0.02	0.839	83.9	0.006
	21 × 10^−5^	61.7 ± 0.1714	244 ± 0.1453	0.88 ± 0.03	0.870	87.0	0.007
Inhibitor (II)	1 × 10^−6^	91.4 ± 0.1412	74.3 ± 0.1241	0.90 ± 0.01	0.572	57.2	0.002
	5 × 10^−5^	86.3 ± 0.1453	110.9 ± 0.1653	0.91 ± 0.01	0.715	71.5	0.003
	9 × 10^−5^	79.6 ± 0.2333	168.4 ± 0.1012	0.92 ± 0.01	0.812	81.2	0.001
	13 × 10^−5^	73.1 ± 0.1453	219.6 ± 0.1893	0.92 ± 0.01	0.856	85.6	0.004
	17 × 10^−5^	64.9 ± 0.1202	330.4 ± 0.1987	0.93 ± 0.01	0.904	90.4	0.007
	21 × 10^−5^	57.6 ± 0.1553	364.3 ± 0.1453	0.95 ± 0.01	0.913	91.3	0.006

### 3.4. EFM studies

**Fig. 5:**
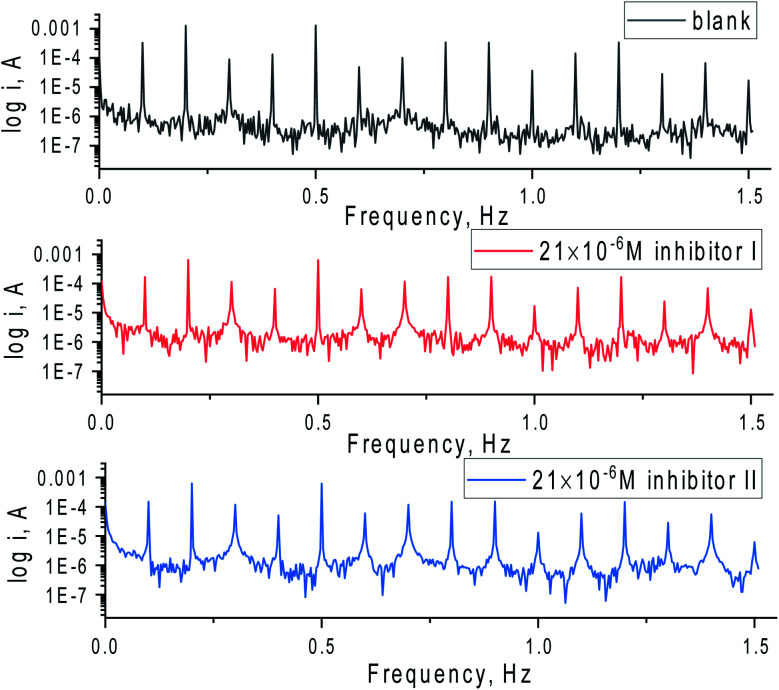
EFM spectra for C-steel in 1 M HCl with and without 21 × 10^−6^ M of the inhibitors (I & II) at 25 °C.

The EFM spectral intermodulation for C-steel in 1 M hydrochloric acid solution before and after adding 21 × 10^−6^ M of the inhibitors (I & II) are displayed in Fig. 5. The bigger peaks were applied to examine *i*_corr_, *β*_c_, *β*_a_, CF-2, and CF-3. The electrochemical factors were concurrently specified and then recorded in Table 5. It can be viewed from this Table 5 that the values of *i*_corr_ decrease in the presence of various doses of 5-arrylidene barbituric acid derivatives than in the presence of only 1 M HCl in the C-steel. The obtained causality factors for the examined data are in excellent quality with their theoretical (2 & 3) values. The order of %IE for 5-arrylidene barbituric acid derivatives achieved from EFM studies is inhibitor II > inhibitor I.

**Table 5:** EFM parameters for C-steel 1 M HCl solution and the presence of altered doses of inhibitors I & II at 25 °C

Comp.	Conc., M	*i*_corr_, μA cm^−2^	*β*_a_, mV dec^−1^	*β*_c_, mV dec^−1^	C.F (2)	C.F (3)	C.R., mmy^−1^	*θ*	IE%
1 M HCl	00	808.5 ± 0.2028	112.9 ± 0.2028	163.7 ± 0.1155	2.09	1.75	370.2		—
Inhibitor (I)	1 × 10^−6^	434.9 ± 0.2431	95.9 ± 0.2134	137 ± 0.1245	2.03	3.3	198.7	0.462	46.2
	5 × 10^−6^	313 ± 0.1452	101.9 ± 0.2354	144 ± 0.1158	2.02	2.36	143.1	0.613	61.3
	9 × 10^−6^	197.5 ± 0.2431	114.4 ± 0.2222	120 ± 0.1447	2.1	3.15	90.2	0.756	75.6
	13 × 10^−6^	141.3 ± 0.2102	110.9 ± 0.2145	115.7 ± 0.2603	1.37	2.18	64.6	0.825	82.5
	17 × 10^−6^	120.7 ± 0.2209	106.3 ± 0.2055	109.1 ± 0.2245	1.44	3.77	55.2	0.851	85.1
	21 × 10^−6^	99.8 ± 0.2010	104.4 ± 0.2218	116.1 ± 0.2403	1.54	1.37	45.6	0.877	87.7
Inhibitor (II)	1 × 10^−6^	327.6 ± 0.1753	147.7 ± 0.1732	149.7 ± 0.2028	2.18	1.98	149.7	0.595	59.5
	5 × 10^−6^	168 ± 0.2028	94.8 ± 0.2309	97.11 ± 0.2245	1.70	2.87	77.1	0.792	79.2
	9 × 10^−6^	140 ± 0.1732	87.9 ± 0.2333	149.5 ± 0.2358	1.93	3.32	64.1	0.827	82.7
	13 × 10^−6^	94.9 ± 0.1453	87.6 ± 0.1202	125.1 ± 0.2475	2.08	3.82	43.4	0.883	88.3
	17 × 10^−6^	85.9 ± 0.2333	129.4 ± 0.1732	152.1 ± 0.2333	1.78	1.27	34.6	0.894	89.4
	21 × 10^−6^	75.7 ± 0.1764	113.7 ± 0.1453	119.4 ± 0.2578	1.35	3.01	39.3	0.906	90.6

### 3.5 Effectiveness of temperature

The impact of the temperature on the rate of corrosion of C-steel in 1 M HCl including a diverse concentration of the investigated inhibitors can be examined *via* the WL method in the temperature ranges from 25 to 55 °C (Table 6). The outcomes showed that by raising the temperature the rate of corrosion rises and declines with dose of these compounds rises for the investigated inhibitors.

**Table 6:** Data of WL measurements for C-steel in 1 M HCl solution with and without altered doses of inhibitors (I & II) at 25–55 °C

Inh.	Conc. (M)	Temp. (°C)	CR (mg cm^−2^ min^−1^)	*θ*	%IE
Inhibitor (I)	Blank (1 M HCl)	25	0.028	—	—
		35	0.033	—	—
		45	0.039	—	—
		55	0.045	—	—
	1 × 10^−6^	25	0.015	0.489	48.9
		35	0.021	0.737	73.7
		45	0.027	0.301	30.1
		55	0.033	0.265	26.5
	5 × 10^−6^	25	0.012	0.608	60.8
		35	0.017	0.483	48.3
		45	0.023	0.408	40.8
		55	0.027	0.386	38.6
	9 × 10^−6^	25	0.010	0.655	65.5
		35	0.014	0.568	56.8
		45	0.019	0.491	49.1
		55	0.024	0.467	46.7
	13 × 10^−6^	25	0.008	0.709	70.9
		35	0.012	0.626	62.6
		45	0.017	0.545	54.5
		55	0.022	0.515	51.5
	17 × 10^−6^	25	0.007	0.742	74.2
		35	0.010	0.689	68.9
		45	0.015	0.621	62.1
		55	0.018	0.592	59.2
	21 × 10^−6^	25	0.006	0.805	80.5
		35	0.008	0.758	75.8
		45	0.012	0.693	69.3
		55	0.014	0.689	68.9
Inhibitor (II)	1 × 10^−6^	25	0.012	0.691	69.1
		35	0.016	0.608	60.8
		45	0.023	0.569	56.9
		55	0.028	0.471	47.1
	5 × 10^−6^	25	0.009	0.739	73.9
		35	0.014	0.655	65.5
		45	0.020	0.638	63.8
		55	0.024	0.558	55.8
	9 × 10^−6^	25	0.007	0.792	79.2
		35	0.011	0.709	70.9
		45	0.017	0.671	67.1
		55	0.022	0.581	58.1
	13 × 10^−6^	25	0.006	0.825	82.5
		35	0.009	0.742	74.2
		45	0.015	0.700	70.0
		55	0.018	0.627	62.7
	17 × 10^−6^	25	0.005	0.876	87.6
		35	0.007	0.810	81.0
		45	0.012	0.754	75.4
		55	0.015	0.686	68.6
	21 × 10^−6^	25	0.003	0.928	92.8
		35	0.005	0.866	86.6
		45	0.009	0.799	79.9
		55	0.011	0.747	74.7

**Fig. 6:**
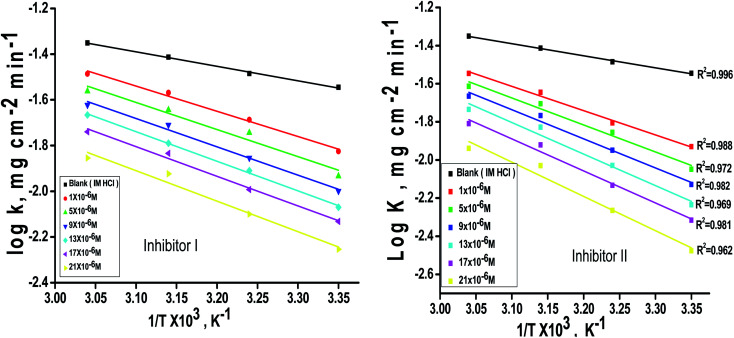
log *k* − 1/*T* curves for C-steel dissolution in 1.0 M HCl in the absence and existence of altered doses of inhibitor (I and II).

**Fig. 7:**
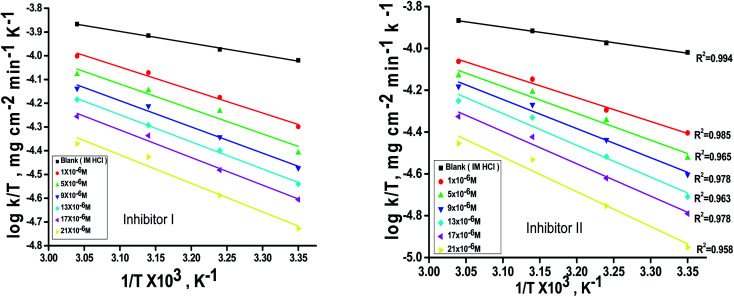
log *k*/*T*–1/*T* curves for C-steel dissolution in 1 M HCl and the presence of altered doses the investigated inhibitors (I & II).

The activation energy 
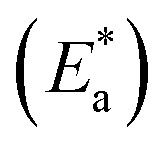
 can be examined by applying Arrhenius equation:10
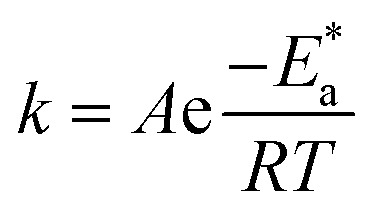
where, *A* is the Arrhenius constant and *k* is the rate of corrosion. Straight lines are displayed in Fig. 6 and their linear regression (*R*^2^) is nearer to 1, and E* can be obtained from the slope. Table 6 displayed that the value of 
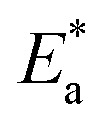
 for the uninhibited solution is lower than that of the inhibited solution, supposing that the dissolution of C-steel is slow within existence of inhibitor.^61^ This is recognized from eqn (10) to be the higher values of 
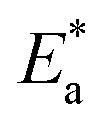
, which cause a lower corrosion rate owing to the construction of the protecting film on the C-steel surface acting as an energy barrier of the C-steel corrosion.^62–64^ Entropy and enthalpy of activation (ΔS*, ΔH*) of the corrosion procedure were determined from the transition state theory.11
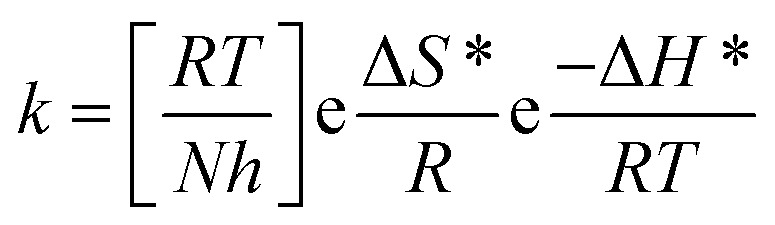
where, *N* is Avogadro's number and *h* is Planck's constant. The graphs of log *k*/*T versus* 1/*T* of C-steel with 1 M hydrochloric acid solution at diverse doses from the examined compounds, provides straight lines as displayed in Fig. 7 for the inhibitors. The thermodynamic parameters are listed in Table 7 shows that the ΔH* values are positive, which signals that the steel dissolution process is endothermic process”. High and negative values of ΔS* show that the activated complex is found in an associated form more than the dissociated form.

**Table 7:** Activation parameters for the dissolution of C-steel in the absence and existence of altered doses of inhibitors (I & II) in 1 M HCl

Inhibitor	Activation parameters				
	Conc., M	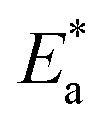 , kJ mol^−1^	ΔH*, kJ mol^−1^	−ΔS*, J mol^−1^ K^−1^	(*R*^2^)
	Blank	12.2 ± 0.1453	9.6 ± 0.1879	108.6 ± 0.2333	0.9941
Inhibitor (I)	1 × 10^−6^	21.1 ± 0.1678	18.6 ± 0.1456	83.5 ± 0.2028	0.9844
	5 × 10^−6^	22.7 ± 0.1478	20.2 ± 0.1453	80.1 ± 0.1453	0.9381
	9 × 10^−6^	23.7 ± 0.1212	21.2 ± 0.2333	78.2 ± 0.1732	0.9834
	1.3 × 10^−5^	24.8 ± 0.1893	21.9 ± 0.2245	77.6 ± 0.2128	0.9969
	1.7 × 10^−5^	24.8 ± 0.2253	22.3 ± 0.2357	77.1 ± 0.1453	0.9849
	2.1 × 10^−5^	25.64 ± 0.1741	23.1 ± 0.2783	67.9 ± 0.1764	0.9563
Inhibitor (II)	1 × 10^−6^	24.4 ± 0.2025	21.8 ± 0.2473	74.8 ± 0.1547	0.9859
	5 × 10^−6^	27.1 ± 0.1732	24.5 ± 0.2214	67.5 ± 0.2264	0.9658
	9 × 10^−6^	29.2 ± 0.1000	26.7 ± 0.2008	64.9 ± 0.2041	0.9785
	1.3 × 10^−5^	31.7 ± 0.1453	29.1 ± 0.2433	62.1 ± 0.1453	0.9636
	1.7 × 10^−5^	32.2 ± 0.1732	29.6 ± 0.2245	55.9 ± 0.1732	0.9787
	2.1 × 10^−5^	34.4 ± 0.1453	31.9 ± 0.2147	51.3 ± 0.2028	0.9562

### 3.6. Adsorption isotherm

Organic compounds inhibit metal corrosion through adsorption on the surface of the metal. The adsorption procedure is considered as a single replacement process of adsorbed water molecules (*x*) by a single inhibitor molecule.^65,66^

12 *I*_(aq)_ + *x*H_2_O_(sur)_ → *I*_(sur)_ + *x*H_2_O_(aq)_

**Fig. 8:**
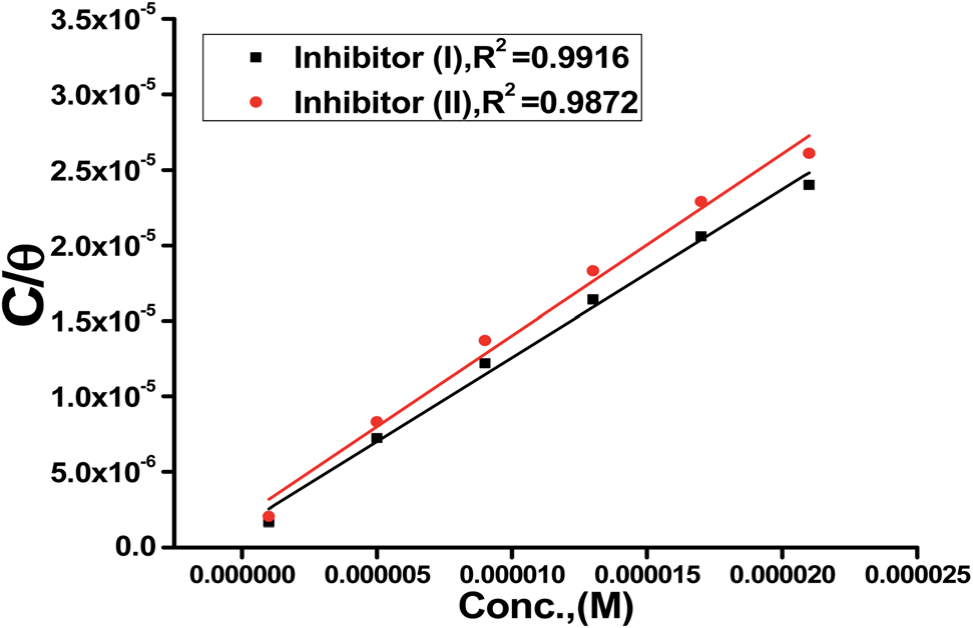
Langmuir isotherm plots for C-steel in 1 M HCl containing various doses of inhibitors (I & II) at 25 °C.

Also, adsorption affords data regarding the interaction between the adsorbed molecules and the surface of the metal. The values of *θ* for diverse doses of the analyzed inhibitors at various temperatures have been applied to describe the most suitable adsorption isotherm to define the adsorption procedure. The outcomes of the studied inhibitors are suitable for the Langmuir adsorption isotherm. Fig. 8 displays the plotting of *C*/*θ versus C* at 25 °C to examine the inhibitors, respectively. The schemes provided straight lines with unit slope, which shows that the adsorption of the examined derivatives on the C-steel surface confirmed the Langmuir equation.^67^13
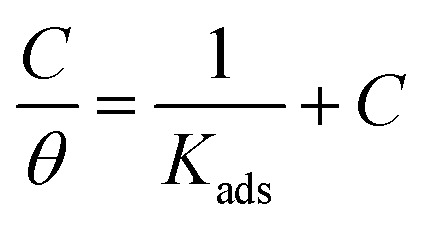
where *C* is the inhibitor concentration and *K*_ads_ is the adsorption equilibrium constant” associated with the free energy of adsorption Δ*G*_ads_ as follows.^68^14
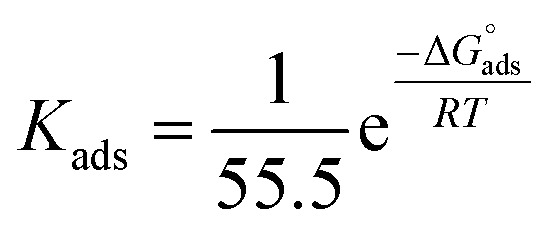
where *T* is the absolute temperature, *R* is the universal gas constant, and 55.5 is the concentration of water on the metal surface in M. The values of *K*_ads_ and 
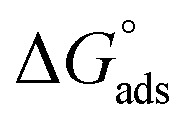
 for 5-arylidene barbituric acid derivatives are listed in Table 8. The increase in the negative value of 
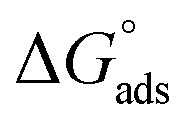
 indicates that these compounds were strongly adsorbed on the C-steel surface in a stable state and that the adsorption process was spontaneous. Furthermore, the values of 
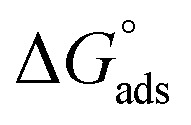
 are −32.5 and −33.0 kJ mol^−1^, which indicates that the adsorption of 5-arylidene barbituric acid derivatives on C-steel is mixed-type, *i.e.*, physisorption and chemisorption, but mainly physisorption because the 
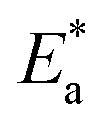
 values increase in the presence of inhibitors than in its absence and %inhibition decreases by increasing the temperature. In addition, the *K*_ads_ values were established to run analogous to the IE% (*K*_II_ > *K*_I_). This result replicates the ability to grow on the metal surfaces due to structural development.^69^

**Table 8:** Equilibrium constant and adsorption free energy of the investigated inhibitors (I & II) adsorbed on C-steel surface at 25 °C

Langmuir isotherm				
Inhibitor	*K* × 10^−5^, M^−1^	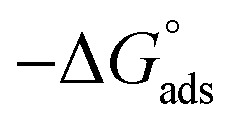 , kJ mol^−1^	Slope	*R* ^2^
Inhibitor (I)	5.07 ± 0.015	32.5 ± 0.1454	1.206	0.9720
Inhibitor (II)	6.97 ± 0.025	33.3 ± 0.2189	1.113	0.9910

### 3.7. DFT studies

**Fig. 9:**
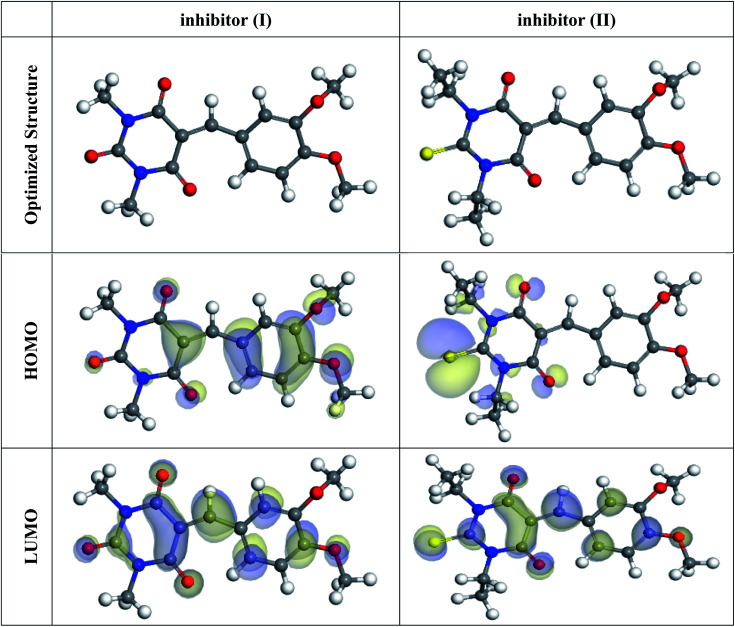
The optimized molecular structures, HOMO, and LUMO for the barbituric acid derivatives using DFT calculations in the aqueous phase.

**Fig. 10:**
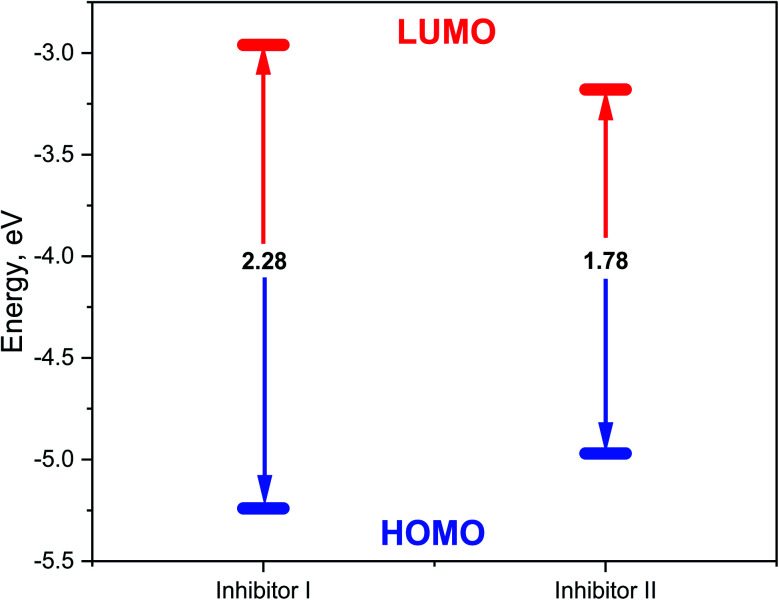
Energy diagram of the frontier molecular orbitals for the investigated inhibitors (I, II) and their assessed Δ*E*.

**Fig. 11:**
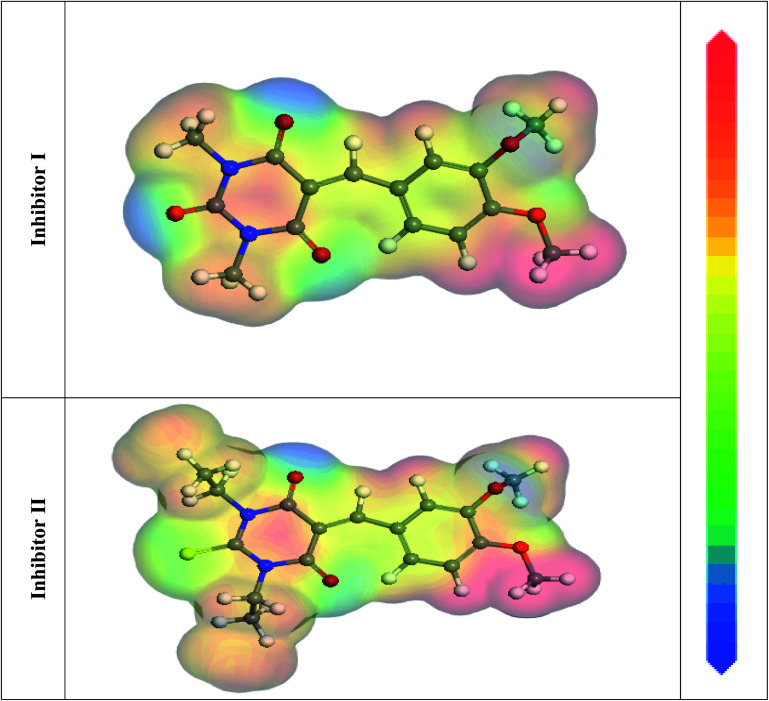
Graphical presentation of the MEP for inhibitors (I & II) using DFT calculations in the aqueous phase.

In the aqueous phase, the optimal structure, HOMO, and LUMO distribution of 5-arylidene barbituric acid derivatives molecules are revealed in Fig. 9, and the quantum chemical characteristics are included in Table 9. Fig. 10 represents the energy diagram of the frontier molecular orbitals for the investigated compounds (I, II) and their assessed Δ*E*. The interaction between the inhibitor molecule and the metal is directed by the HOMO and LUMO energies, according to the frontier orbital theory.^70^*E*_HOMO_ signifies the capability of a molecule to contribute electrons and *E*_LUMO_ signifies the capacity of a molecule to receive electrons.^71^ As a result, the corrosion inhibition capability of an inhibitor molecule with high *E*_HOMO_ and low *E*_LUMO_ values improves. Similarly, high corrosion protection efficiency was proposed for an inhibitor molecule with a low energy gap between the LUMO and HOMO energy (Δ*E*) since proffering an electron from *E*_HOMO_ to *E*_LUMO_. As given in Table 9, compound II has a larger *E*_HOMO_ value of −4.97 eV as related to compound I. As shown in Fig. 9, for the 5-arylidene barbituric acid derivatives molecules, we notice that the HOMO level is pinpointed on the phenyl, methoxy, and pyrimidine moieties, implying that the O and N atoms are the desired location for electrophilic attacks on the surface of C-steel. This would enhance the adsorption capability of 5-arylidene barbituric acid derivatives molecules on the C-steel surface and therefore enhance the protection efficiency, which is in concurs excellently with the empirical results. Moreover, the *E*_LUMO_ values are −1.48 eV for compound II (Table 9) lower than those of compound I, indicating the great inhibition efficacy for compound II. Similarly, the energy gap (Δ*E*) is another critical aspect in approving the inhibitor molecule's corrosion prevention capability, which improves as the (Δ*E*) value decreases.^72^ Compound II exhibits lower (Δ*E*) values (1.78 eV) than compound I, as shown in Table 8, indicating a higher propensity for compound II to be adsorbed on the C-steel surface. Furthermore, because of the low electronegativity (*χ*), the 5-arylidene barbituric acid derivatives molecules have a high potential reactivity to offer electrons to the metal surface.^73^ Furthermore, the global hardness *η* and softness *σ* of a molecule are important qualities that determine its consistency and reactivity. Because electrons are smoothly afforded to the C-steel surface *via* adsorption, soft molecules are more reactive compared to hard molecules.^74^ The Δ*N* values determine the electron contributing capability of the inhibitors, and the higher the Δ*N* value, the larger the electron providing facility of the inhibitor molecule. According to Lukovits's study,^75^ when Δ*N* <3.6, the %IE improves with greater electron donating ability. Based on the calculated values of Δ*N* as listed in Table 9, the greater the Δ*N* values for compound II (1.64) than compound I. This means that compound II molecule has greater tendency to offer electrons to the surface of C-steel, as related to compound I. Furthermore, the dipole moment is an important indicator for forecasting the path of corrosion protection.^76^ The augmentation in the dipole moment leads to an increase in the deformation energy and better molecule adsorption on steel surface, enhancing the inhibitory activity.^77^ Compound II has a greater dipole moment value (7.95 debye) than compound I, as shown in Table 9, indicating a strong tendency for compound II to be adsorbed on the C-steel surface and enhance the inhibition effectiveness. Furthermore, the molecular size of the 5-arylidene barbituric acid derivatives and their tendency to protect the C-steel surface in corrosive environment have a clear relationship. The inhibition efficiency increases with increasing molecular structure size because of the contact area between the surfactant's molecules and the steel surface raise.^78^ As mentioned in Table 9, compound II demonstrates a greater area (396.36 Å^2^); for this purpose, they have greater inhibition proficiency than compound I. MEP mapping is a powerful 3D vision tool for distinguishing the net electrostatic effect established over a molecule from total charge dispersal.^79^ The red colors in Fig. 10 signify the highest electron density, with MEP being the biggest negative (nucleophilic reaction). The blue colors, on the other hand, signify the most positive region (electrophilic reaction).^80^ The largest negative (red color) regions in methoxy and pyrimidine moieties are generally over N and O atoms, whereas the lower density (green color) regions in 5-arylidene barbituric acid derivative molecules are mostly over the phenyl moieties. In the keto form, MEP, on the other hand, showed the most positive (blue hue) area over oxygen. The locations in 5-arylidene barbituric acid derivative molecules with the highest electron density may be the most proper for interactions with the C-steel surface. Fig. 11 represents the graphical presentation of MEP for inhibitors (I & II) using DFT calculations in the aqueous phase.

**Table 9:** Calculated quantum chemical parameters for the structure of inhibitors (I & II) in the aqueous phase

Compound	Inhibitor (I)	Inhibitor (II)
*E*_HOMO_, eV	−5.24	−4.97
*E*_LUMO_, eV	−2.96	−3.18
Δ*E*, eV	2.28	1.78
*I*, eV	5.24	4.97
*A*, eV	2.96	3.18
*χ*, eV	4.10	4.08
*η*, eV	1.14	0.89
*σ*, eV	0.88	1.12
Δ*N*, eV	1.27	1.64
Dipole moment, Debye	7.77	7.95
Molecular surface area, Å^2^	316.86	396.36

### 3.8. MC simulations

**Fig. 12:**
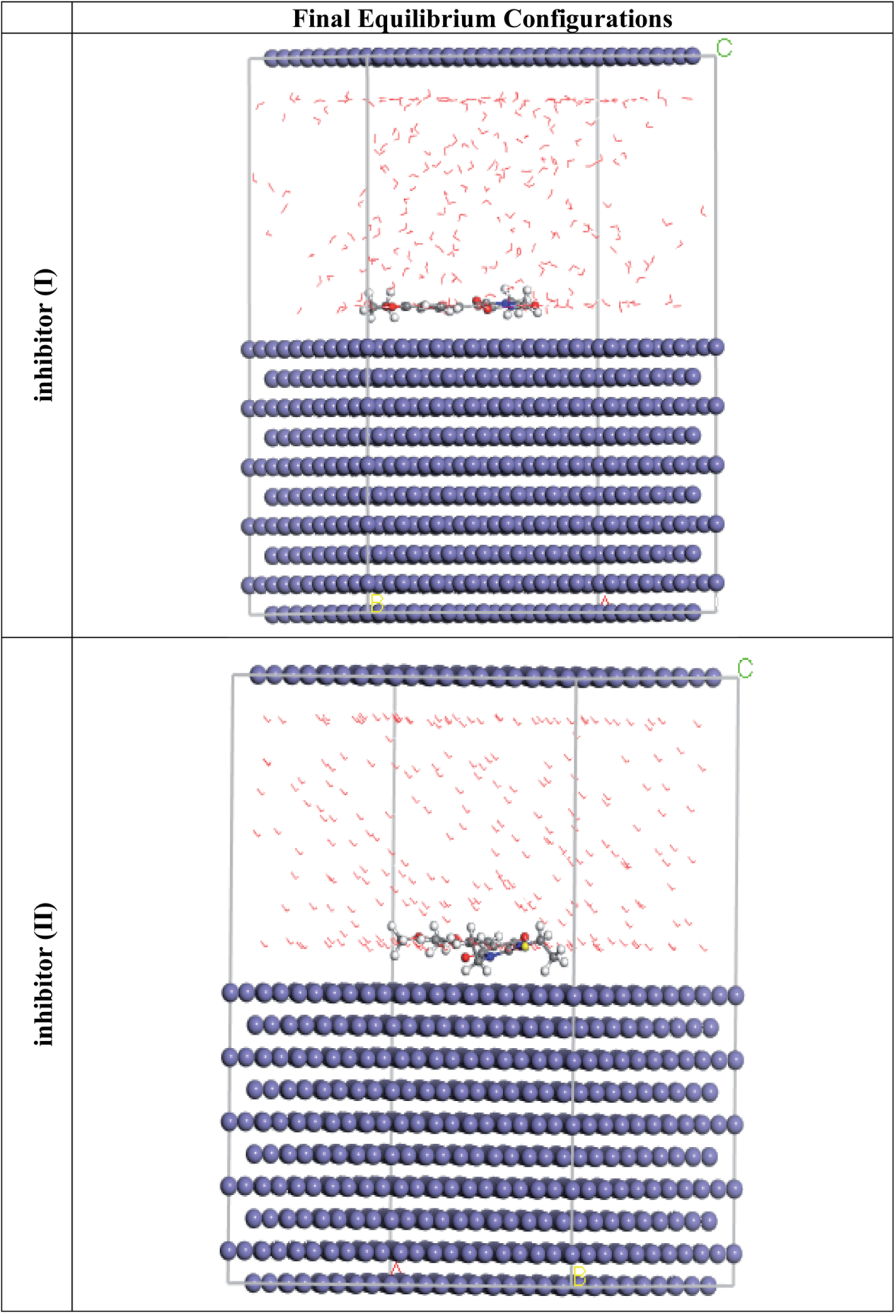
The most suitable adsorption configuration for the barbituric acid derivatives on the Fe (110) substrate obtained from the adsorption locator module.

MC simulations are theoretical approaches for comprehending the nature of the interaction between the 5-arylidene barbituric acid derivative molecules and the C-steel surface thru the adsorption procedure by retaining the adsorption locator module. Therefore, Fig. 12 divulges the highest appropriate adsorption configurations for the 5-arylidene barbituric acid derivatives molecules on the C-steel surface, which is located in nearly parallel or flat disposition, showing an increase in the scope of adsorption and greatest surface coverage.^81^Table 10 also lists the results of the Monte Carlo simulation, including the adsorption energy for the relaxed adsorbate molecules, the rigid adsorption energy for unrelaxed adsorbate molecules, and the deformation energy for the relaxed adsorbate molecules.^82^Table 10 shows that compound II (−3512.49 kcal mol^−1^) has a higher adsorption energy than compound I (−3490.57 kcal mol^−1^), implying that compound II has a strong adsorption on the C-steel surface, creating stationary adsorbed layers that protect the C-steel from corrosion, which concurs with the empirical results. Furthermore, the findings in Table 10 divulge that the adsorption energies of compound II (unrelaxed and relaxed) are higher than those of compound I before and after the geometry optimization process, indicating that compound II has a higher inhibitory efficiency than compound I. When one of the adsorbate is abolished, the d*E*_ads_/d*N*_i_ values explain the energy of the metal-adsorbate configuration.^83^ The d*E*_ads_/d*N*_i_ value for compound II (−224.14 kcal mol^−1^) is higher than that of compound I molecules (204.29 kcal mol^−1^), indicating that compound II molecules have better adsorption than compound I molecules. Furthermore, the d*E*_ads_/d*N*_i_ values for water are close to −14.26 kcal mol^−1^, which is low compared with the values for 5-arylidene barbituric acid derivatives, indicating that 5-arylidene barbituric acid derivatives molecules have a more durable adsorption than water molecules, indicating that water molecules can be replaced by 5-arylidene barbituric acid derivatives molecules. As a result, the 5-arylidene barbituric acid derivative molecules are forcefully adsorbed on the C-steel surface and form a robust adsorbed defensive layer, resulting in a corrosion shield for the C-steel surface in destructive conditions, as demonstrated by both empirical and theoretical research.

**Table 10:** Data and descriptors calculated by the Monte Carlo simulation (MC) for the adsorption of the barbituric acid derivatives on iron (110)

Structures	Adsorption energy/kcal mol^−1^	Rigid adsorption energy/kcal mol^−1^	Deformation energy/kcal mol^−1^	d*E*_ads_/d*N*_i_: inhibitor kcal mol^−1^	d*E*_ads_/d*N*_i_: water kcal mol^−1^
Fe (110)	−3490.57	−3664.63	174.06	−204.29	−14.39
Inhibitor I					
Water					
Fe (110)	−3512.49	−3688.35	175.86	−224.14	−14.18
Inhibitor II					
Water					

### 3.9 Surface examination (SEM & EDX analysis)

**Fig. 13:**
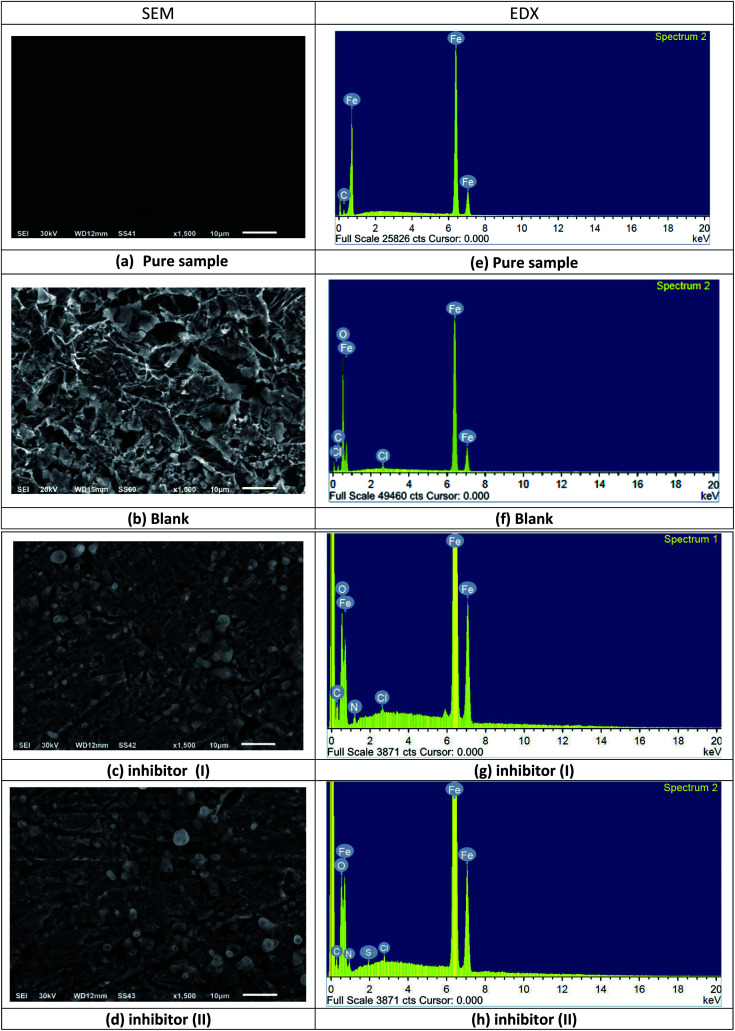
SEM images and EDX spectra of the C-steel surface before and after immersion in 1 M HCl in the absence and presence of 21 × 10^−6^ M compound I and II for 24 h at 25 °C (SEM images: (a) is pure sample, (b) is blank, (c) is inhibitor I, (d) is inhibitor II) and in (EDX images: (e) is pure sample, (f) is blank, (g) is inhibitor I, (h) is inhibitor II).

Fig. 13a–d describes the C-steel samples in 1 M HCl in the absence and presence of 21 × 10^−6^ M compound I and II. The SEM image of the pristine C-steel (Fig. 13a) exhibits a moderately smooth surface. On the other hand, after the exposure of C-steel to 1 M HCl for 24 h, the C-steel interfaces were severely scratched and destroyed (Fig. 13a). However, after adding an optimum dose of compound I and II, the surface turns smoother and free slightly from the corrosion product; this shows the protective action of the inhibitors through restraining the active centers of the C-steel surface. Fig. 13e–h implies the EDX analysis and the atomic content percentage of uninhibited and inhibited samples, respectively. The strong Fe signal (Fig. 13e) indicated a Fe-rich pristine C-steel surface. However, untreated C-steel surface exposed to 1 M HCl as a corrosive medium exhibited O, Cl, and Fe signals (Fig. 13f). This might be related to strong corrosion and/or formation of iron chloride and/or iron oxide layers on the CS surface (Fig. 13f). As revealed in Fig. 13d and g, the EDX spectrums of compound I and compound II display additional signals owing to the occurrence of N and S. The occurrence of N and S elements in the EDX patterns of the inhibited surface shows that the inhibitor molecule is adsorbed on the C-steel interface and inhibits its corrosion (Table 11).

**Table 11:** Atomic content percentage of the C-steel surface before and after immersion in 1 M HCl in the absence and presence of 21 × 10^−6^ M compound I and II for 24 h at 25 °C

Atomic content percentage	Fe	C	Cl	O	S	N
Free	**92.16**	**7.84**	—	—	—	—
Blank	**63.05**	**11.69**	**2.35**	**22.91**	—	—
Inhibitor I	**71.5**	**10.59**	**1.86**	**15.57**	—	**0.48**
Inhibitor II	**72.03**	**11.35**	**1.53**	**14.31**	**0.25**	**0.53**

### 3.10 Mechanism of adsorption and inhibition

The adsorption of inhibitor on the steel surface can be used to suggest an inhibitory mechanism. In general, a single adsorption mode between the inhibitor and the metal surface is impractical due to the complicated nature of adsorption and inhibition of a specific inhibitor. Based on the chemical structures of 5-ABAs, they may adsorb on the active site of a C-steel surface in the current system. As a result, the inhibitory phenomenon may be affected by the following adsorption:

(i) Because of the neutral O atoms in 5-ABAs, they may be protonated in an acid solution as: (5-ABAs) + *x*H^+^ → [5-ABAsH]^*x*+^

As a result, 5-ABAs exists as [5-ABAsH]^*x*+^ in acidic solutions because Cl^−^ may adsorb on the metal surfaces,^39^ they provide an excess negative charge in the solution, favoring cation adsorption”. The negatively charged metal surface may absorb [5-ABAsH*x*]^*x*+^. In other words, there might be a synergistic relationship between the adsorbed Cl^−^ and protonated inhibitor.^40^

(ii) In addition to physical adsorption, 5-ABAs can be adsorbed on metal surfaces using the chemisorption mechanism, which involves the formation of coordinate bonds between the lone electron pairs of the O and S atoms and the empty orbital of Fe atoms, strengthening the combination in tension between the inhibitor molecule and the electrode surface.

(iii) It is widely believed that the heterocyclic ring is the primary adsorption center of heterocyclic compounds. Because of the heterocyclic ring, 5-ABAs contain a lot of p-electrons, and they may be adsorbed on the metal surface with the donor–acceptor interactions between the p-electrons of the heterocyclic ring and the unoccupied d-orbitals of Fe.

(iv) Derivative II is more efficient than derivative I due to: (a) derivative II has higher molecular size than derivative I as it may cover a larger area from the C-steel surface. (b) Derivative II has S atom instead of O atom in their structures and S atom is more basic than O atom, *i.e.*, it may donate more electron pairs than O atoms and (c) derivative II has ethyl group instead of methyl group in derivative I.

## 4 Conclusions

(1) 5-Arylidene barbituric acid derivatives establish a very good inhibition for C-steel in HCl solution.

(2) 5-Arylidene barbituric acid derivatives inhibit the C-steel corrosion by adsorption on its surface and make the layer film.

(3) The inhibition efficiency of these derivatives increases by increasing their doses.

(4) The adsorption of these derivatives on C-steel in HCl solution applied by Langmuir isotherm.

(5) The values of *C*_dl_ decline and *R*_ct_ rise compared to the blank solution when the inhibitors are added, confirming the adsorption of inhibitor molecules on the surface of C-steel.

(6) The polarization data indicated that these derivatives behave as mixed type inhibitors.

(7) DFT calculation and MC simulations were achieved to demonstrate the adsorption sites found in the inhibitor's molecules.

(8) Surface analysis was confirmed using the SEM and EDX techniques.

(9) There is a good agreement between the experimental and theoretical studies.

## Conflicts of interest

The authors declare that there is no conflict of interest between them and any body else.

## Supplementary Material
